# Structure-From-Motion in 3D Space Using 2D Lidars

**DOI:** 10.3390/s17020242

**Published:** 2017-02-03

**Authors:** Dong-Geol Choi, Yunsu Bok, Jun-Sik Kim, Inwook Shim, In So Kweon

**Affiliations:** 1 Robotics and Computer Vision Lab, KAIST, 291 Daehak-ro, Yuseong-gu, Daejeon 34141, Korea; dgchoi@rcv.kaist.ac.kr (D.-G.C.); iwshim@rcv.kaist.ac.kr (I.S.); iskweon@kaist.ac.kr (I.S.K.); 2 Broadcasting Media Research Lab, Electronics and Telecommunications Research Institute, 218 Gajeong-ro, Yuseong-gu, Daejeon 34129, Korea; 3 Center for Robotics Research, Korea Institute of Science and Technology, Seoul 02792, Korea; junsik.kim@kist.re.kr

**Keywords:** 2D lidar, structure-from-motion, pose estimation

## Abstract

This paper presents a novel structure-from-motion methodology using 2D lidars (Light Detection And Ranging). In 3D space, 2D lidars do not provide sufficient information for pose estimation. For this reason, additional sensors have been used along with the lidar measurement. In this paper, we use a sensor system that consists of only 2D lidars, without any additional sensors. We propose a new method of estimating both the 6D pose of the system and the surrounding 3D structures. We compute the pose of the system using line segments of scan data and their corresponding planes. After discarding the outliers, both the pose and the 3D structures are refined via nonlinear optimization. Experiments with both synthetic and real data show the accuracy and robustness of the proposed method.

## 1. Introduction

Two-dimensional lidars (Light Detection And Ranging) have been one of the most important sensors in many robotic applications because of its accuracy and robustness at measuring distance. It provides two-dimensional distance measurements on its own scanning plane. Many robotic systems [1,2,3,4] that require localization or even simultaneous localization and mapping (SLAM) have successfully utilized 2D lidars as their essential sensor. In recent years, many low-cost depth cameras have become popular (e.g., Microsoft Kinect, Intel RealSense, etc.) and have been used for various applications, such as photometric vision [5,6], object recognition [7,8] and odometry estimation [9,10,11]. However, 2D lidars are still preferred because of their long range and robustness to lighting conditions.

Compared to depth cameras, one of the key drawbacks of 2D lidars is that it provides only two-dimensional distance measurements at a time. It usually consists of a laser range finder and a rotating mirror to measure distances of scene points on a scan plane. Two-dimensional localization and mapping [12] is a popular application of 2D lidars, which is useful for wheeled robots and ground vehicles. It works based on the assumption that the motion of the lidar is two-dimensional, that the measurements have been collected at a consistent height. Once the assumption is broken due to the tilting motion of the lidar, there is no way to estimate the six degree-of-freedom (DOF) motion of the lidar.

Several approaches have been proposed to handle 2D lidars in a 3D space. The first approach is to fuse another motion information with the lidar measurements. Many systems utilize inertial measurement units (IMUs) to compensate for the tilting motion of the sensor [13,14,15]. However, this sensor fusion mostly aims to make the measurements of the tilted sensor match the 2D map, so that the compensated measurements can be used for 2D applications.

The second and most preferred solution is to build a 3D lidar system. One 2D lidar on a rotating stage provides 3D measurements around the sensor [16,17,18]. This 3D scanning device becomes an essential sensor for outdoor and automated systems such as self-driving cars [19,20,21,22]. Most of the successful systems participating in the defense advanced research projects agency (DARPA) urban challenge [23], which requires self-driving capability in urban environments, have been equipped with the 3D lidar system. Zhang and Singh [24] rotated a 2D lidar to obtain depth information. The advantage of their work is that the whole 3D structure is captured in a static pose, while the proposed system must move. To use their system, however, the motor must also be calibrated with high accuracy because their work assumes that the rotation speed is known. Moreover, their algorithm has a probability of divergence in the optimization process because it considers the system’s motion during a single 3D scan.

The final approach is to use visual information along with the lidar measurements [25]. Because vision sensors provide abundant information about the scene, fusion of the visual information and the distance measurements by the 2D lidars enable 6D pose estimation. Pose estimation using cameras is related to ‘structure-from-motion (SFM)’, which is one of most popular issues in the area of computer vision. However, it requires many distinguishable visual features. Camera-based SFM fails if there are few visual features due to homogeneous areas.

In this paper, we propose a novel methodology of estimating the 6D pose of a system that consists of only 2D lidars. It is basically similar to the SFM framework, but is purely based on the measurements of 2D lidars. Instead of visual features corresponding to a 3D point cloud, we utilize the line segments of the scan data corresponding to the planar structures of a target scene. The pose of the system is initialized and refined using a number of the line–plane correspondences, and the 3D structures of the target scene are also updated via a nonlinear refinement process. The proposed algorithm is verified using real data. It successfully estimated the 3D structures and the 6D poses of the sensors without any additional sensors or motion constraint.

## 2. Overview of the Proposed Method

**Figure 1:**
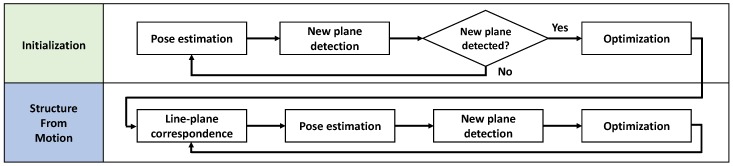
Flow chart of the proposed method.

The flow chart of the proposed method is shown in Figure 1. It is divided into two steps, the initialization step and the SFM step. In the first step, we assume that the parameters of the three planes scanned by 2D lidars are known. Line-plane correspondences are designated manually, and the sensor pose is estimated using the correspondences (Section 3). The pose estimation is iterated until new planes are detected (Section 4.2). If we detect a new plane, we refine both the sensor poses and plane parameters by minimizing the squared sum of the Euclidean distances between the scan data and the corresponding plane (Section 4.3). The SFM process after the initialization step is similar to that of the initialization step, except the designation of line-plane correspondences is done automatically using the previous pose information (Section 4.1). After initializing the sensor pose, both the sensor poses and the plane parameters are refined via nonlinear optimization, even when a new plane is not detected.

## 3. Sensor Pose Estimation

This section presents the algorithm of estimating the pose of a sensor system that consists of 2D lidars. It is assumed that the system is fully calibrated. The sensor system and its own coordinate system are referred to as the ‘sensor’ and ‘sensor coordinate system’, respectively, for the rest of this paper.

### 3.1. Pose Parameterization Using Two Lines on Two Planes

In this section, we show that the sensor pose is represented with two lines on two planes. Let us assume that we have a plane in the world coordinate system with known parameters, $\Pi ={\left[u^{⊤}\;d\right]}^{⊤}$, where u and *d* denote the unit normal vector and the constant term of the plane, respectively. If it is scanned by a 2D lidar, the intersecting line of the plane and the lidar’s scan plane appears in the scan data. The line can be defined by two points $p^{\prime }$ and $q^{\prime }$ in the sensor coordinate system. They are also represented as $p=Rp^{\prime }+t$ and $q=Rq^{\prime }+t$ in the world coordinate system, where [Rt] is the sensor-to-world transformation, i.e., the sensor pose. The direction vector v≡p−q of the line in the world coordinate system becomes simply

(1) $$v=Rv^{\prime },$$

where $v^{\prime }\equiv p^{\prime }-q^{\prime }$ is the direction vector of the line in the sensor coordinate system.

The points p and q must lie on the plane Π, and this gives

(2) $$\begin{array}{cc} & \Pi^{⊤}\left[\begin{array}{c} p\\ 1 \end{array}\right]=u^{⊤}\left(Rp^{\prime }+t\right)+d=0, \end{array}$$

(3) $$\begin{array}{c} u^{⊤}R\left(p^{\prime }-q^{\prime }\right)=u^{⊤}Rv^{\prime }=0. \end{array}$$

Once we have multiple line–plane correspondences and the known rotation R, the translation t is simply estimated using Equation (2) via a linear estimation. Thus, we focus on the estimation of the rotation matrix R.

We first show that the rotation R can be decomposed into the two parts, one computed using the direction vectors in the world coordinate system and the other computed using the direction vectors in the sensor coordinate system.

**Figure 2:**
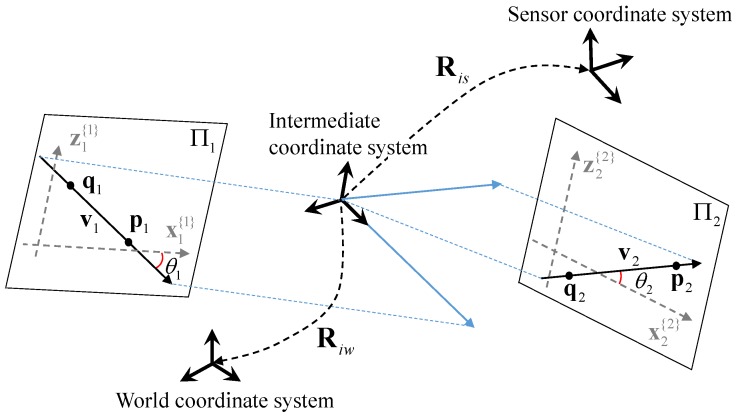
Parameterization of rotation matrix using two line angles, $\theta_{1}$ and $\theta_{2}$, on the scene planes. The sensor-to-world rotation matrix R is expressed as two rotation matrices $R_{iw}$ and $R_{is}$. The rotation matrix $R_{iw}$ is only dependent on $\theta_{1}$ and $\theta_{2}$. From the sensor measurement, $R_{is}$ is already known as well as the direction vector $v_{1}^{\prime }$ and $v_{2}^{\prime }$.

Let us assume that we have two scan lines $l_{1}$ and $l_{2}$ corresponding to two planes $\Pi_{1}$ and $\Pi_{2}$, respectively, as shown in Figure 2. The direction vector of $l_{n}$ is defined as $v_{n}$ in the world coordinate system, and $v_{n}^{\prime }$ in the sensor coordinate system. It should be noted that the plane parameters $\Pi_{n}$ are defined only in the world coordinate system.

We set an intermediate coordinate system, whose *x*-*z* plane is equal to the plane spanned by $v_{1}$ and $v_{2}$, and its *x*-axis coincides with $v_{1}$. The rotation $R_{iw}$ from the intermediate coordinate system to the world coordinate system becomes

(4) $$R_{iw}=\left[\begin{array}{ccc} r_{iw}^{1} & r_{iw}^{2} & \left(r_{iw}^{1}\times r_{iw}^{2}\right) \end{array}\right],$$

where

(5) $$r_{iw}^{1}=\frac{v_{1}}{|v_{1}|},\;\;r_{iw}^{2}=\frac{v_{1}\times v_{2}}{|v_{1}\times v_{2}|}.$$

Similarly, the rotation $R_{is}$ from the intermediate coordinate to the sensor coordinate is

(6) $$R_{is}=\left[\begin{array}{ccc} r_{is}^{1} & r_{is}^{2} & \left(r_{is}^{1}\times r_{is}^{2}\right) \end{array}\right],$$

where

(7) $$r_{is}^{1}=\frac{v_{1}^{\prime }}{|v_{1}^{\prime }|},\;\;r_{is}^{2}=\frac{v_{1}^{\prime }\times v_{2}^{\prime }}{|v_{1}^{\prime }\times v_{2}^{\prime }|}.$$

Thus, the rotation R from the sensor coordinate system to the world coordinate system is simply

(8) $$R=R_{iw}R_{is}^{⊤}.$$

Note that $R_{iw}$ is dependent only on $v_{n}$, while $R_{is}$ is only dependent on $v_{n}^{\prime }$. From the sensor measurements, $R_{is}$ is already known as well as the direction vectors $v_{n}^{\prime }$. To estimate the rotation matrix completely, we need only two corresponding direction vectors $v_{1}$ and $v_{2}$ in the world coordinate system.

Now, we represent the direction vectors $v_{n}$ in terms of angles $\theta_{n}$, which will be referred to as ‘line angles’ for the rest of this paper. We define the coordinate system of each plane $\Pi_{n}$ so that its *y*-axis coincides with the plane normal $u_{n}$. Then, the direction vector in the plane coordinate system can be expressed as

(9) $$v_{n}^{\left\{n\right\}}={\left[\begin{array}{ccc} \cos\theta_{n} & 0 & \sin\theta_{n} \end{array}\right]}^{⊤},$$

where $v_{n}^{\left\{k\right\}}$ denotes the direction vector $v_{n}$ in the $\Pi_{k}$-coordinate system. The direction vector $v_{n}$ in the world coordinate system is equal to $v_{n}=R_{\Pi_{n}}v_{n}^{\left\{n\right\}}$, where $R_{\Pi_{n}}$ is the fixed rotation matrix from the $\Pi_{n}$-coordinate system to the world coordinate system. Until now, the rotation estimation from the sensor to the world coordinate systems are parameterized with two line angles, one for $v_{1}^{\left\{1\right\}}$ and the other for $v_{2}^{\left\{2\right\}}$ on two different planes.

One may wonder how to set up the plane coordinate system with just the plane normal. Of course, there is one-dimensional freedom to choose the *x*- and *z*-axis. It can be set arbitrarily. Because we compute the line angle on the plane coordinate system, different selections of coordinate axes induce a different line angle matching the coordinate selection. Thus, the estimated rotation is invariant to the selection of plane coordinate systems.

In the following section, we will derive how to estimate the line angle $\theta_{n}$ in the scene plane.

### 3.2. Estimation of the Line Angles on the Planes

As mentioned in Section 3.1, the sensor-to-world transformation is obtained if two line angles $\theta_{1}$ and $\theta_{2}$ in each plane coordinate system are known. To estimate the angles, we use the invariant property of angle between direction vectors: the angle between two direction vectors in the sensor coordinate system should be preserved in the world or plane coordinate system. The inner product $\alpha_{2}$ between two direction vectors is simply measurable in the sensor coordinate system and is calculated in $\Pi_{2}$-coordinate system as

(10) $$\alpha_{3}={v_{2}^{\prime }}^{⊤}v_{1}^{\prime }=v_{2}^{\{2\}⊤}\left(Av_{1}^{\left\{1\right\}}\right),$$

where A is a rotation matrix from $\Pi_{1}$-coordinate to $\Pi_{2}$-coordinate, i.e., $A=R_{\Pi_{2}}^{⊤}R_{\Pi_{1}}$, which is known.

**Figure 3:**
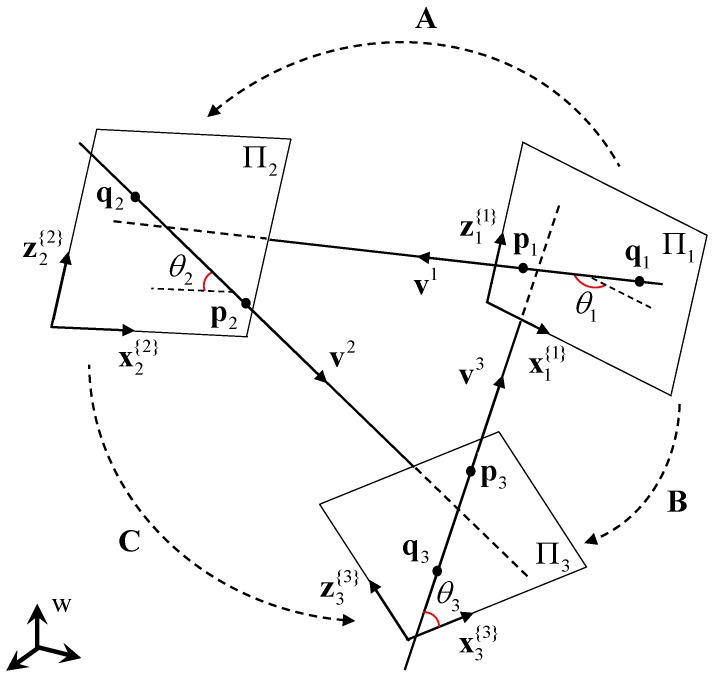
Three lines on three planes. From the three known angles among the lines, we drive three equations of $\theta_{1}$, $\theta_{2}$ and $\theta_{3}$. A, B and C indicate the known rotation matrices from $\Pi_{1}$- to $\Pi_{2}$-coordinate system, from $\Pi_{1}$- to $\Pi_{3}$-coordinate system, and from $\Pi_{2}$- to $\Pi_{3}$-coordinate system, respectively.

Equation (10) contains two unknown variables $\theta_{1}$ and $\theta_{2}$ in $v_{1}^{\left\{1\right\}}$ and $v_{2}^{\left\{2\right\}}$, respectively. Thus, it is not possible to solve it directly with only a single constraint Equation (10). This is resolved by introducing another line $l_{3}$ on plane $\Pi_{3}$. By adding one line, we have one more unknown $\theta_{3}$ and two more constraint equations from the in-between angles as

(11) $$\begin{array}{c} \alpha_{2}={v_{3}^{\prime }}^{⊤}v_{1}^{\prime }=v_{3}^{\{3\}⊤}\left(Bv_{1}^{\left\{1\right\}}\right), \end{array}$$

(12) $$\begin{array}{c} \alpha_{1}={v_{3}^{\prime }}^{⊤}v_{2}^{\prime }=v_{3}^{\{3\}⊤}\left(Cv_{2}^{\left\{2\right\}}\right), \end{array}$$

where $B=R_{\Pi_{3}}^{⊤}R_{\Pi_{1}}$ and $C=R_{\Pi_{3}}^{⊤}R_{\Pi_{2}}$ are the rotation matrices from $\Pi_{1}$- to $\Pi_{3}$-coordinate system and from $\Pi_{2}$- to $\Pi_{3}$-coordinate system, respectively. Now, we have three trigonometric equations with three unknowns. The geometric relation is shown in Figure 3.

The elements of A, B and C at the *i*-th row and the *j*-th column are denoted by $a_{ij}$, $b_{ij}$ and $c_{ij}$, respectively. Equations (10) and (11) are expressed in terms of $\theta_{n}$:

(13) $$\begin{array}{c} k_{1}c_{2}+k_{2}s_{2}=\alpha_{2}, \end{array}$$

(14) $$\begin{array}{c} k_{3}c_{3}+k_{4}s_{3}=\alpha_{3}, \end{array}$$

where we defined that

(15) $$\begin{array}{c} c_{n}\equiv \cos\theta_{n}, \end{array}$$

(16) $$\begin{array}{c} s_{n}\equiv \sin\theta_{n}, \end{array}$$

(17) $$\begin{array}{c} k_{1}\equiv a_{11}c_{1}+a_{13}s_{1}, \end{array}$$

(18) $$\begin{array}{c} k_{2}\equiv a_{31}c_{1}+a_{33}s_{1}, \end{array}$$

(19) $$\begin{array}{c} k_{3}\equiv b_{11}c_{1}+b_{13}s_{1}, \end{array}$$

(20) $$\begin{array}{c} k_{4}\equiv b_{31}c_{1}+b_{33}s_{1}. \end{array}$$

From Equation (13) and $c_{2}^{2}+s_{2}^{2}=1$, we obtain $c_{2}$ and $s_{2}$ as functions of $\theta_{1}$:

(21) $$\begin{array}{c} c_{2}=\frac{\alpha_{2}k_{1}\pm k_{2}\sqrt{k_{1}^{2}+k_{2}^{2}-\alpha_{2}^{2}}}{k_{1}^{2}+k_{2}^{2}}, \end{array}$$

(22) $$\begin{array}{c} s_{2}=\frac{\alpha_{2}k_{2}\mp k_{1}\sqrt{k_{1}^{2}+k_{2}^{2}-\alpha_{2}^{2}}}{k_{1}^{2}+k_{2}^{2}}. \end{array}$$

Similarly, we derive $c_{3}$ and $s_{3}$ as functions of $\theta_{1}$ from Equation (14) and $c_{3}^{2}+s_{3}^{2}=1$:

(23) $$\begin{array}{c} c_{3}=\frac{\alpha_{1}k_{3}\pm^{\prime }k_{4}\sqrt{k_{3}^{2}+k_{4}^{2}-\alpha_{3}^{2}}}{k_{3}^{2}+k_{4}^{2}}, \end{array}$$

(24) $$\begin{array}{c} s_{3}=\frac{\alpha_{1}k_{4}\mp^{\prime }k_{3}\sqrt{k_{3}^{2}+k_{4}^{2}-\alpha_{3}^{2}}}{k_{3}^{2}+k_{4}^{2}}. \end{array}$$

The sets of double signs, (±, ∓) and ($\pm^{\prime }$, $\mp^{\prime }$), are independent. We substitute Equations (21)–(24) into Equation (12)

(25) $$k_{11}-k_{12}=\mp \left(\pm^{\prime }2\left(k_{7}k_{8}+k_{9}k_{10}\right)\sqrt{k_{5}k_{6}}\right),$$

where we define that

(26) $$\begin{array}{c} k_{5}\equiv k_{1}^{2}+k_{2}^{2}-\alpha_{2}^{2}, \end{array}$$

(27) $$\begin{array}{c} k_{6}\equiv k_{3}^{2}+k_{4}^{2}-\alpha_{1}^{2}, \end{array}$$

(28) $$\begin{array}{c} k_{7}\equiv \alpha_{3}\left(c_{11}k_{2}k_{3}-c_{13}k_{1}k_{3}+c_{31}k_{2}k_{4}-c_{33}k_{1}k_{4}\right), \end{array}$$

(29) $$\begin{array}{c} k_{8}\equiv \alpha_{2}\left(c_{11}k_{1}k_{4}+c_{13}k_{2}k_{4}-c_{31}k_{1}k_{3}-c_{33}k_{2}k_{3}\right), \end{array}$$

(30) $$\begin{array}{c} k_{9}\equiv \alpha_{3}\alpha_{2}\left(-c_{11}k_{1}k_{3}-c_{13}k_{2}k_{3}-c_{31}k_{1}k_{4}-c_{33}k_{2}k_{4}\right)+\alpha_{1}\left(k_{1}^{2}+k_{2}^{2}\right)\left(k_{3}^{2}+k_{4}^{2}\right), \end{array}$$

(31) $$\begin{array}{c} k_{10}\equiv c_{11}k_{2}k_{4}-c_{13}k_{1}k_{4}-c_{31}k_{2}k_{3}+c_{33}k_{1}k_{3}, \end{array}$$

(32) $$\begin{array}{c} k_{11}\equiv k_{5}k_{7}^{2}+k_{6}k_{8}^{2}, \end{array}$$

(33) $$\begin{array}{c} k_{12}\equiv k_{9}^{2}+k_{5}k_{6}k_{10}^{2}. \end{array}$$

Squaring both sides of Equation (25) to remove the root sign, we obtain a 16th order equation with two variables, $c_{1}$ and $s_{1}$. For convenience of calculation, we change the equation to a 16th order polynomial equation of a single variable. We multiply the squared Equation (25) by $1/s_{1}^{16}$, and every term of the equation can be expressed as

(34) $$\begin{array}{c} h{\left(\frac{c_{1}}{s_{1}}\right)}^{\sigma_{1}}{\left(\frac{1}{s_{1}^{2}}\right)}^{\sigma_{2}},\\ (\sigma_{1}\geq 0,\sigma_{2}\geq 0,\sigma_{1}+2\sigma_{2}=16), \end{array}$$

where we defined that the *h* is a coefficient value, and the $\sigma_{1}$ and the $\sigma_{2}$ are integer values. Substituting $1+(c_{1}^{2}/s_{1}^{2})$ to $1/s_{1}^{2}$, the squared Equation (25) is expressed as a 16th order polynomial equation of $c_{1}/s_{1}$:

(35) $$\begin{array}{c} h{\left(\frac{c_{1}}{s_{1}}\right)}^{\sigma_{1}}{\left(1+\frac{c_{1}^{2}}{s_{1}^{2}}\right)}^{\sigma_{2}}=h{\left(\frac{c_{1}}{s_{1}}\right)}^{\sigma_{1}}+h{\left(\frac{c_{1}}{s_{1}}\right)}^{\sigma_{1}+2\sigma_{2}}. \end{array}$$

From the 16th order polynomial equation, we obtain 16 candidates of $c_{1}/s_{1}$, which is equal to $1/\tan\theta_{1}$, within the range from −π/2 to π/2. Because $\tan(\theta_{1}+\pi )$ is equal to $\tan\theta_{1}$, the number of candidates is doubled. They are substituted into Equations (21)–(24) to calculate $\theta_{2}$ and $\theta_{3}$. Finally, we obtain combinations of the three angles, $\theta_{1}$, $\theta_{2}$, and $\theta_{3}$ that satisfy Equation (3) and Equation (12).

### 3.3. Physical Constraint for Solution Selection

From the solutions of the rotation matrix R that were calculated in Section 3.2, we obtain solutions of the translation vector t using a least square method with singular value decomposition (SVD)

(36) $$\left[\begin{array}{c} u_{n}^{⊤}\;u_{n}^{⊤}Rp_{n}^{\prime }+d_{n}\\ u_{n}^{⊤}\;u_{n}^{⊤}Rq_{n}^{\prime }+d_{n} \end{array}\right]\left[\begin{array}{c} t\\ 1 \end{array}\right]=0.$$

All the estimated poses R and t satisfy the given conditions Equation (3) and Equations (10)–(12), but there exist physically non-realizable poses. To choose only physically realizable poses, we adopt a new physical constraint that the location of the sensor must be in front of all planes.

A plane divides the 3D space into two sides, a front side and a back side. In our implementation, we set the normal directions of planes as their back side (the sign of $d_{n}$ is also determined simultaneously). Since the sensor should be located only in front of the planes, the inner product of the plane normals and the sensor position vector must be negative:

(37) $$\Pi_{n}^{⊤}t<0.$$

Multiple pose candidates may meet this condition, and, in our experience, there are two physically realizable poses in most cases. It is impossible to distinguish which one is true. We can further choose the true six-dimensional sensor pose using an additional line–plane correspondence or an assumption that the pose of the sensor does not change much compared to the previous one, in the case of continuous sequences.

The whole process of the proposed method is described in Algorithm 1.

**Algorithm 1** 6-DOF Pose Estimation of a Lidar System using Three Lines**INPUT** (a) Six points on the three scan lines (two in each line) in the sensor coordinate system (b) Parameters of three planes in the world coordinate system (c) One-to-one correspondences between scan lines and planes. **OUTPUT:** Sensor-to-world transformation, R and t 1. Compute $\alpha_{n}$ using three line vector, $v_{n}^{\left\{n\right\}}=p_{n}^{\prime }-q_{n}^{\prime }$, in the sensor coordinate system. (*n* = 1–3) 2. Compute three rotation matrices A, B, and C using plane parameters in the world coordinate system. 3. Compute the coefficients of $k_{1}$ to $k_{12}$. 4. Compute the coefficients of 16th order polynomial equation using $k_{1}$ to $k_{12}$. 5. Solve the equation to obtain candidates of $\theta_{1}$ 6. Obtain combinations of $\theta_{1}$, $\theta_{2}$ and $\theta_{3}$ from Equations (21) to (24). 7. Extract combinations of $\theta_{1}$, $\theta_{2}$ and $\theta_{3}$ that satisfy Equation (3) and Equation (12). 8. Determine solutions of the sensor-to-world transformation, R and t, which meet the conditions mentioned in Section 3.3.

## 4. Structure from Motion

### 4.1. Line-Plane Correspondence

As we mentioned before, the line-plane correspondences in the initialization step are designated manually. After the initialization, the correspondences are designated automatically using the previous pose information. A new scan line obtained from the sensor system can be defined by two points, $p^{\prime }$ and $q^{\prime }$, in the sensor coordinate system. We transform the points into the world coordinate system using the previous pose $\left[R_{pre}\;t_{pre}\right]$, which gives

(38) $$\begin{array}{cc} \hat{p} & =R_{pre}p^{\prime }+t_{pre},\\ \hat{q} & =R_{pre}q^{\prime }+t_{pre}. \end{array}$$

We compute the distance between two points and every plane and find the minimum value $l_{min}$:

(39) $$\begin{array}{c} l_{min}=\min_{i=1∼M}\sqrt{{\left(u_{i}^{⊤}\hat{p}+d_{i}\right)}^{2}+{\left(u_{i}^{⊤}\hat{q}+d_{i}\right)}^{2}}, \end{array}$$

where *M* is the number of planes. If $l_{min}$ is smaller than a user-defined threshold, we designate the pair of the line and the plane as a line-plane correspondence. Lines with $l_{min}$ bigger than the threshold do not correspond to any plane. They will be referred to as ‘non-assigned lines’ for the rest of this paper.

### 4.2. New Plane Detection

The non-assigned lines are accumulated as we repeat the structure-from-motion process. If the number of non-assigned lines is bigger than a user-defined number, we detect new planes using these lines. In this paper, the user-defined number was used as 30, which was determined empirically. We select two lines to generate a plane candidate and measure distances between the candidate and the remaining non-assigned lines. Those with the distance smaller than a user-defined threshold are classified as inliers of the plane candidate. Among the plane candidates generated by all combinations of non-assigned lines, we determine one with the largest number of inliers as a new plane.

### 4.3. Nonlinear Optimization

All variables in the whole sequence are refined via nonlinear optimization. The cost function *f* for the optimization is the squared sum of the Euclidean distances between the plane and lidar points in the world coordinate system

(40) $$f\left(\Pi_{1}∼\Pi_{M},R_{1}∼R_{N},t_{1}∼t_{N}\right)=\sum_{i=1}^{M}\sum_{j\in A_{i}}\sum_{p\in B_{i,j}}\Pi_{i}^{⊤}\left(R_{j}p+t_{j}\right),$$

where *N* is the number of scans of sensor data. $A_{i}$ is the set of pose indices that scan the plane $\Pi_{i}$, and $B_{i,j}$ is the set of points that lie on the plane $\Pi_{i}$ and belong to the *j*-th pose. We adopt the Levenberg-Marquardt [26] algorithm for the nonlinear optimization.

The refinement may be achieved by conventional registration methods such as iterative closest point (ICP) [27,28]. However, the nonlinear optimization is better than the registration because of three reasons listed below:

- the correspondence between points and planes are not changed in the optimization process;
- the overlap between a single scan and a point cloud is very narrow (several lines on two scanning planes);
- the nonlinear optimization utilizes Jacobian while the registration does not.

## 5. Experimental Results

We evaluated the performance of the proposed pose estimation and SFM algorithms through several experiments. In every experiment, the error of an estimated pose T is defined as a residual transformation $\delta T=T_{ref}^{-1}T$ between the reference transformation $T_{ref}$ and the estimated pose. Rotation error is measured by the rotation angle of Rodrigues’ rotation formula [29] of the rotation in the residual transformation, and the translation error is measured by the translation part of δT.

### 5.1. Evaluation of Pose Estimation Algorithm

**Figure 4:**
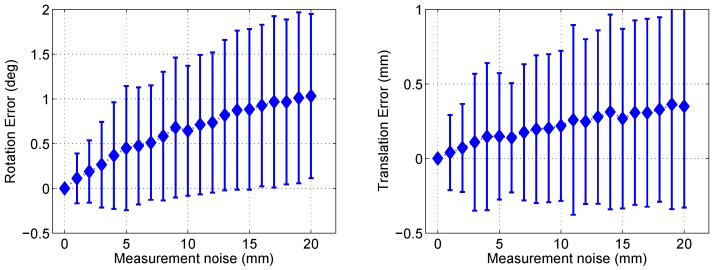
Experimental results using synthetic data. The value *x* of ‘Measurement noise’ (horizontal axis) means that the noise added to the points are generated in the interval of [−x,x].

We performed two experiments that use synthetic and real data to evaluate the performance of the proposed pose estimation algorithm. For the synthetic experiment, we generate a set of data that consists of a sensor-to-world transformation, three plane parameters and six points on the planes. The transformation is generated randomly with its maximum rotation angle as 50 degrees and maximum translation as four meters along every axis. The plane parameters are also generated randomly with a maximum distance of five meters from the world origin. The points are generated in the interval of [−4,4] meters along every axis from the world origin. Two of the three coordinates are generated randomly while the other is computed using the plane parameters to generate points ‘on the planes’. We added white noise with varying intervals to the points and compared the result by the proposed algorithm to the ground truth of the sensor-to-world transformation. We performed 1000 trials and computed the mean and standard deviation of the rotation and translation error, shown in Figure 4. The measurement noise in the horizontal axis indicates the interval in which the noise is generated. Both the rotation error and translation error increase as the measurement noise increases. Although the proposed algorithm does not utilize many points to reduce the effect of noise, the result is not very sensitive to the noise of scan data. In real-world cases, moreover, techniques such as line fitting may reduce the effect of noise.

**Figure 5:**
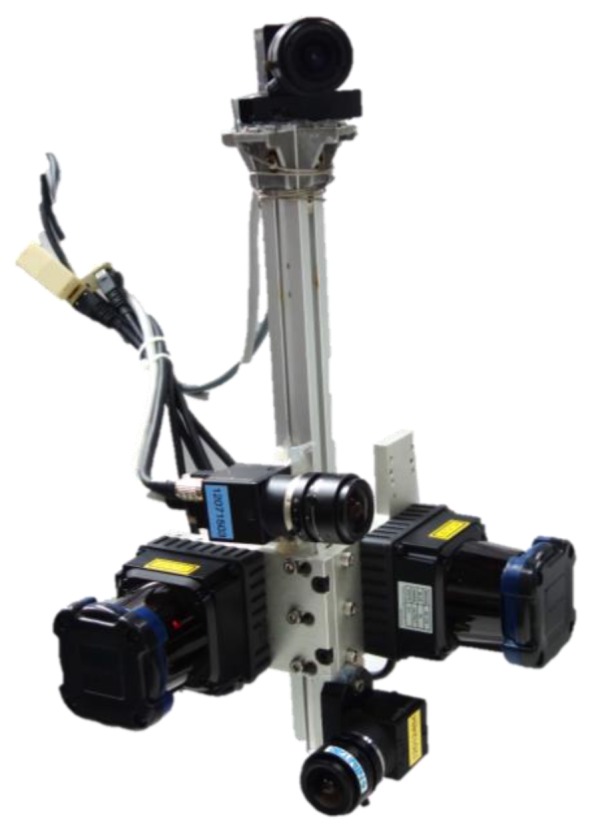
Hand-held sensor system for experimental validations. It consists of two 2D lidars headed to the ceiling and three cameras headed to the side of the lidars and heading to the direction of the system. We use only two 2D lidars for pose estimation. The cameras are attached to obtain color information. The maximum scan rate of the system is about 20 Hz. The system is fully calibrated, including both intrinsic and extrinsic parameters.

For the real data experiment, we designed a hand-held sensor system that consists of two 2D lidars (UTM-30LX, Hokuyo, Osaka, Japan) and three cameras (Flea3, PointGery, Richmond, BC, Canada, 640×480 pixels). The lidars are pointed at the ceiling, and the two cameras are pointed at the side of the lidars (see Figure 5), and one camera is pointed at the forward direction of the system. The cameras are only used to acquire color information. The maximum scan rate of the system is about 20 Hz. The system is fully calibrated: camera intrinsic parameters using [30], lidar to camera [31,32] and between two lidars [33].

**Figure 6:**
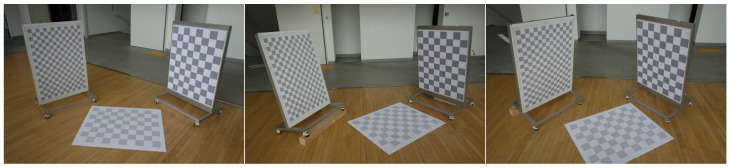
Three non-parallel planes in three different configurations are captured by the sensor system in Figure 5.

We captured three non-parallel checkerboard patterns in three different configurations (see Figure 6) to test the performance of the proposed method in various scene structures. For each configuration, the relative poses among the planes are computed using a number of images captured by two cameras with known intrinsic parameters. The ground plane is scanned by both lidars, while the other two are captured by each lidar and camera. The images captured by two cameras are used to compute the ‘reference’ relative poses between the planes and the sensor system. Among all the scans, we selected 351 scans whose images do not include serious motion blur (the sensor was in motion while the data is captured). The projection errors of the checkerboard corners in the selected scans are smaller than one pixel.

**Figure 7:**
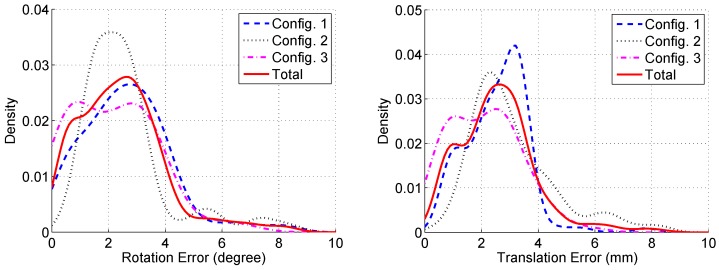
Distributions of the pose difference between the estimated and the reference poses.

The distribution of the difference between the poses computed using laser scans (proposed) and checkerboard images (reference) are displayed in Figure 7, which is estimated by the kernel density estimation method. We show the error distributions of the three different configurations. Most scans have rotation errors smaller than four degrees and translation errors smaller than four millimeters. The mean and standard deviation values of the pose errors are shown in Table 1. The experimental results shows that the proposed method performs robustly in various plane configurations.

### 5.2. Evaluation of Structure-from-Motion Algorithm

**Figure 8:**
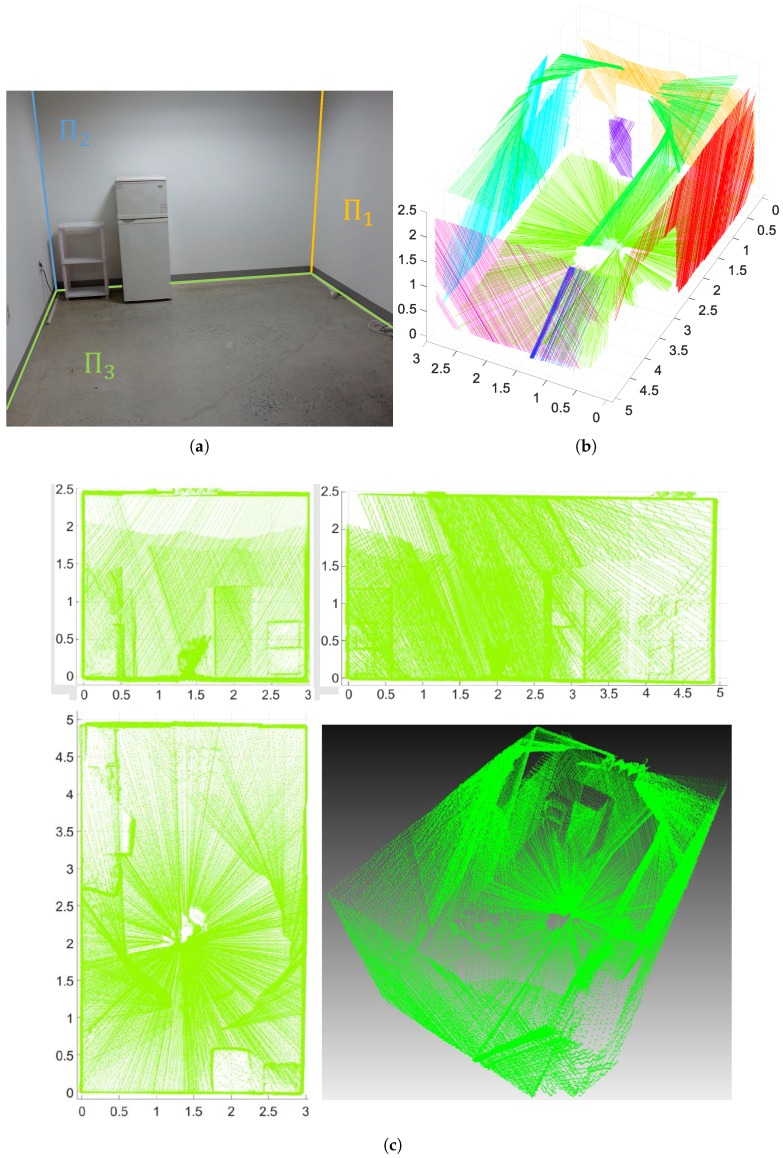
3D reconstruction results of the proposed method: (**a**) real environment (**b**) scanned line segments displayed in colors of corresponding planes; and (**c**) 3D reconstruction results by accumulating scan data.

The proposed SFM algorithm can be used in many applications, especially localization and mapping. All processes except data collection (i.e., motion estimation, structure-from-motion, and nonlinear-optimization) were performed offline. We set two geometric parameters for the experiments. Only lines longer than 0.5 m were used for line extraction with lidar’s scan points, and the inlier bound of the plane was set at 0.05 m. These parameters are determined empirically. For the first experiment, we scanned a small room using the sensor system continuously and estimated the pose of each scan. As shown in Figure 8a, three perpendicular planes, $\Pi_{1}$, $\Pi_{2}$ and $\Pi_{3}$ are used as references for the initialization step. It should be noted that we extracted four lines from two lidar scans—lines corresponding to $\Pi_{1}$ and $\Pi_{3}$ from one lidar, and lines corresponding to $\Pi_{2}$ and $\Pi_{3}$ from the other lidar—to determine a unique solution for each scan, as mentioned in Section 3.3. In Figure 8b, scanned line segments are displayed in colors of corresponding planes. The results shown in Figure 8c verifies that the proposed method gives accurate pose information of the system. Six-hundred-and-fifty scans are accumulated and eight planes are detected based on the poses to reconstruct the 3D structure of the room.

The size of the room measured from the reconstruction result is given in Table 2, and compared to that measured by a laser distance meter (GLM 80, Bosch, Stuttgart, Germany).

**Figure 9:**
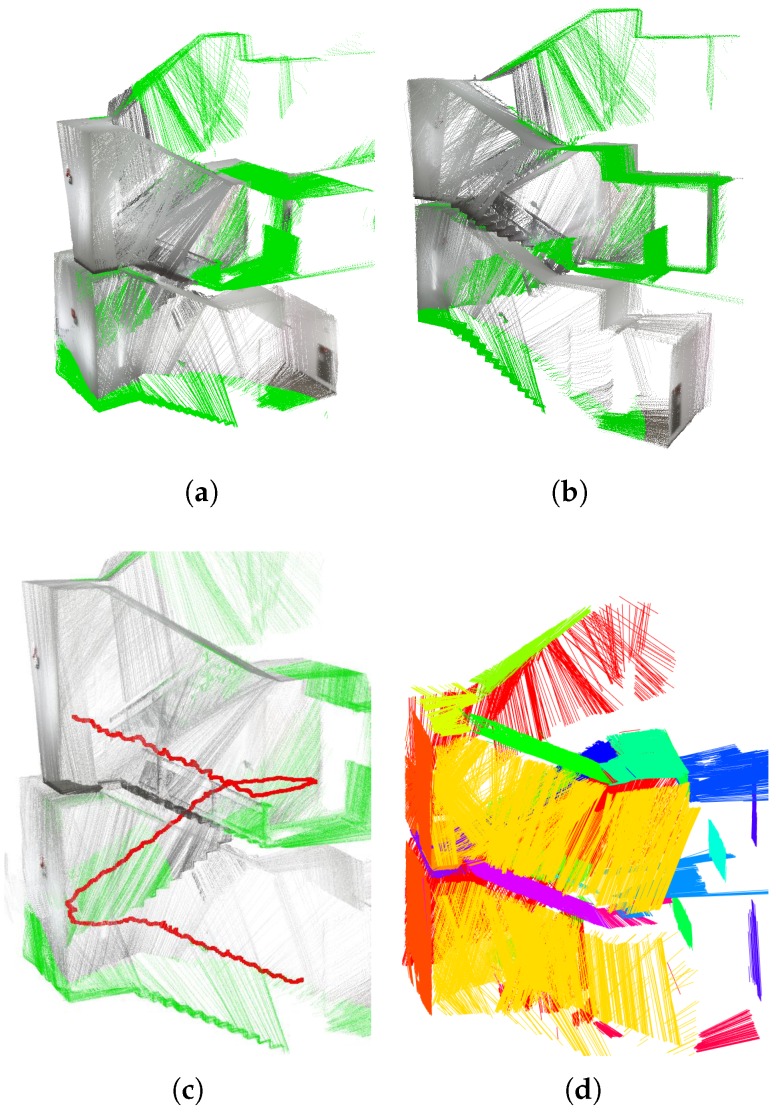
Reconstruction results: (**a**,**b**) 3D reconstruction results using the proposed method; (**c**) estimated trajectory (red dots) of the sensor system with 3500 scans down the stairs; and (**d**) scanned line segments displayed in colors of corresponding planes.

**Figure 10:**
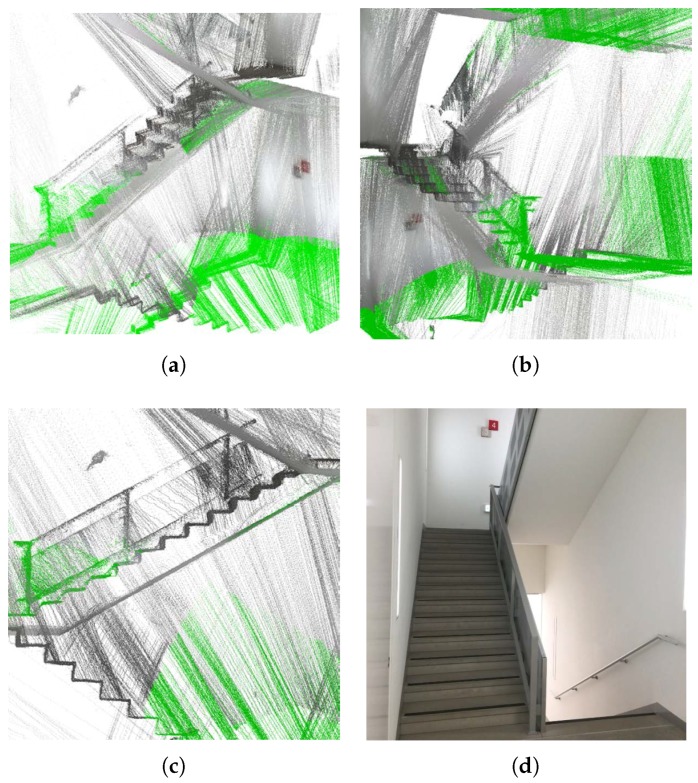
Reconstruction results: (**a**–**c**) detailed views of the result with color information; (**d**) real environment.

For the second experiment, we captured scan data while we passed through a stairway. Figure 9 shows the 3D reconstruction result of the target scene. As shown in Figure 10d, the environment is very challenging for vision based methods because it does not contain enough visual features. However, the proposed method does not suffer from the homogeneous areas because it requires only structural information, not visual features. Thus, it gives an accurate 3D reconstruction result as shown in Figure 9a,b. In Figure 9c, the locations of the sensor system (red dots) are overlaid on the 3D reconstruction result. Scanned line segments are also displayed in colors of corresponding planes, in Figure 9d. In this experiment, we detected 23 planes in total. The steps are not detected as planes because the stairs are scanned vertically – the scanning plane was perpendicular to the longer side of the stairs so that lines on them were shorter than the threshold (0.5 meters in our experiment). In Figure 10, we obtained color information of scan data from the images just for visualization. As shown in Figure 10a–c, we can recognize the shapes of both the handrail and the stairs. In this experiment, the sensor system moved about 25 m (down the stairway) in 125 s while it captured 3500 frames of scan data. This demonstrates the potential of the proposed method in lidar-only mapping and navigation.

## 6. Conclusions

In this paper, we have presented a novel structure-from-motion methodology using 2D lidars. The proposed algorithm uses only 2D range data, without any additional sensors. We have proposed a pose estimation method using three line-plane correspondences. To estimate the line angles, we have used the angles between measured lines and derived a 16th polynomial equation. We have also proposed a new structure-from-motion process using 2D lidar data. After discarding outliers, both the pose and the 3D structures were refined via nonlinear optimization. The experiments using real data validate that the proposed algorithm provides accurate and robust results in real environments.

There are several works that could improve the proposed system and method. The proposed system uses two 2D lidars to obtain three lines that lie on each different plane using a minimal number of sensors. We will try several sensor configurations to find the optimal line information. In this paper, we performed 3D reconstructions using the proposed method, but the result can be more accurate using recent loop closure techniques on scene matching. Additional sensors, such as GPS, and IMU, or even cameras, can be attached to improve the accuracy of the motion estimation.

## Figures and Tables

**Table 1:** The pose errors in different plane configurations.

Configuration	Number of Scans	Rotation Error	Translation Error
		Mean(std) in Degree	Mean(std) in mm
1	113	2.6431 (1.5471)	2.5639 (1.0834)
2	108	2.6104 (1.5523)	3.0944 (1.5391)
3	130	2.1617 (1.4839)	2.1124 (1.2203)
Total	351	2.4548 (1.5379)	2.5599 (1.3458)

**Table 2:** The results of the room measurement.

(Unit: mm)	Width	Length	Height
Laser distance meter	2973.1	4918.7	2374.7
Proposed	2957.6	4924.1	2371.65
Error	15.5 (0.521%)	5.4 (0.110%)	3.05 (0.128%)
